# Can adjuvant immune checkpoint inhibitors improve the long-term outcomes of hepatocellular carcinoma with high-risk recurrent factors after liver resection? A meta-analysis and systematic review

**DOI:** 10.3389/fonc.2024.1374262

**Published:** 2024-05-24

**Authors:** Lingbo Hu, Yenan Kong, Yingli Qiao, Aidong Wang

**Affiliations:** ^1^ Department of Hepatopancreatobiliary Surgery, Taizhou Hospital of Zhejiang Province Affiliated to Wenzhou Medical University, Taizhou, Zhejiang, China; ^2^ Department of Hepatopancreatobiliary Surgery, Enze Hospital, Taizhou Enze Medical Center (Group), Taizhou, Zhejiang, China

**Keywords:** hepatocellular carcinoma, immune checkpoint inhibitors, prognosis, liver resection, adjuvant therapy

## Abstract

**Background:**

Administering adjuvant therapy following liver resection is crucial for patients with hepatocellular carcinoma (HCC) exhibiting high-risk recurrence factors. Immune checkpoint inhibitors (ICIs) are effective against unresectable HCC; however, their effectiveness and safety for this specific patient group remain uncertain.

**Methods:**

We conducted an extensive literature search across four scholarly databases to identify relevant studies. Our primary endpoints were overall survival (OS), recurrence-free survival (RFS), and adverse events (AEs). OS and RFS were quantified using hazard ratios (HRs), whereas the 1-, 2-, and 3-year OS and RFS rates were expressed as risk ratios (RRs). Additionally, the incidence of AEs was calculated.

**Results:**

Our meta-analysis included 11 studies (N = 3,219 patients), comprising two randomized controlled trials (RCTs) and nine retrospective studies. Among these, eight studies reported HRs for OS, showing a statistically significant improvement in OS among patients receiving adjuvant ICIs (HR, 0.60; 95% confidence interval [CI], 0.45–0.80; p < 0.0001). All included studies reported HRs for RFS, indicating a favorable impact of adjuvant ICIs (HR, 0.62; 95% CI, 0.52–0.73; p < 0.0001). Moreover, aggregated data demonstrated improved 1- and 2-year OS and RFS rates with adjuvant ICIs. The incidence rate of AEs of any grade was 0.70 (95% CI, 0.49–0.91), with grade 3 or above AEs occurring at a rate of 0.12 (95% CI, 0.05–0.20).

**Conclusion:**

Adjuvant ICI therapy can enhance both OS and RFS rates in patients with HCC exhibiting high-risk recurrence factors, with manageable AEs.

**Systematic review registration:**

https://www.crd.york.ac.uk/prospero/#recordDetails PROSPERO, identifier CRD42023488250.

## Introduction

Liver cancer ranks sixth in global cancer prevalence and is the third leading cause of cancer-related mortality (1). Hepatocellular carcinoma (HCC), accounting for approximately 90% of primary liver cancer cases, dominates in incidence (2). Liver resection is the primary treatment for HCC (2); however, the notable recurrence rate following liver resection significantly affects patient prognosis (3), particularly in cases with high-risk recurrence factors such as tumor size exceeding 5 cm, presence of multiple tumors, satellite nodules, microvascular invasion (MVI), and portal vein tumor thrombus (PVTT), all of which substantially elevate the risk of early recurrence (4–10). Therefore, integrating adjuvant therapies is crucial for reducing postoperative recurrence risk in these patients (11). Several adjuvant therapies, including transarterial chemoembolization (TACE), hepatic arterial infusion chemotherapy, and sorafenib, have demonstrated efficacy (12–14).

Although immune checkpoint inhibitors (ICIs) are recognized as effective therapies for unresectable HCC (15–17), their role as adjuvant therapy after HCC resection is debatable (18, 19). This meta-analysis aims to clarify the efficacy of ICIs as adjuvant treatment following liver resection in patients with HCC exhibiting high-risk recurrence factors.

## Methods

This systematic review is registered in PROSPERO (registration no. CRD42023488250).

### Search strategy

Comprehensive searches were conducted on four primary databases—Web of Science, PubMed, Embase, and the Cochrane Library—up to November 30, 2023, with an update of the search results on April 20, 2024. The search terms comprised a combination of MeSH terms and keywords, including “hepatocellular carcinoma,” “immune checkpoint inhibitors,” “immunotherapy,” and “adjuvant therapy.” Detailed search strategies for each database are presented in **Supplementary Material S1**.

### Inclusion and exclusion criteria

The inclusion criteria comprised the following (1): Studies, including randomized controlled trials (RCTs) and non-RCTs, that investigated the comparative outcomes of adjuvant ICIs versus no adjuvant ICIs in patients with HCC exhibiting high-risk recurrence factors (2); studies on interventions involving ICI monotherapy or their combination with TACE or tyrosine kinase inhibitors (TKIs), considered as adjuvant ICI interventions (3); studies providing data on at least one primary outcome measure, such as overall survival (OS) or recurrence-free survival (RFS). The exclusion criteria were as follows: (1) Studies involving patients lacking high-risk recurrence factors; (2) studies comparing various combinations of ICIs with other treatment modalities; (3) non-comparative studies, abstracts, case reports, and review articles.

### Definitions

OS was defined as the duration from the date of surgical intervention to mortality, whereas RFS was defined as the period from the date of surgery until tumor reappearance. OS and RFS were primary endpoints analyzed as time-to-event outcomes. The 1-, 2-, and 3-year OS and RFS rates indicated the proportion of patients who remained alive or free from tumor recurrence at these intervals after liver resection, respectively. Adverse events (AEs) were assessed following the guidelines outlined in the National Cancer Institute Common Terminology Criteria for Adverse Events (CTCAE), version 5.0.

### Quality assessment and data extraction

Initial quality assessment and data extraction were conducted by two independent investigators. The Newcastle-Ottawa Scale (NOS) assessed the quality of nonrandomized comparative trials, with scores categorized as low (≤5 points), medium (6–7 points), and high (≥8 points) (20). The Cochrane risk of bias tool was employed to assess potential biases in each study (21). Customized, structured forms were employed for data extraction, including the first author’s name, publication year, patient demographics, and tumor characteristics, as well as primary outcomes, including OS; RFS; the 1-, 2-, and 3-year OS and RFS rates; and AEs. In cases of discordance, a third researcher was consulted to achieve consensus.

### Statistical analysis

Hazard ratios (HRs) and corresponding 95% confidence intervals (CIs) were calculated using the inverse variance method. Risk ratios (RRs) and corresponding 95% CIs were computed via the Mantel–Haenszel method. Incidence rates of AEs of any grade and those graded 3 or higher were also computed with corresponding 95% CIs. Heterogeneity among the studies was assessed using the Q statistic and I^2^ index, with I^2^ values of 25% and 50% indicating low and moderate heterogeneity, respectively. Depending on the observed level of heterogeneity, the appropriate test model was employed; specifically, a random-effects model was employed when I^2^ exceeded 50% (20). Sensitivity analysis was conducted to validate the robustness of the findings. Publication bias was evaluated using funnel plots. Subgroup analyses were planned based on variables such as study design, patient age, tumor characteristics (size, number), presence of MVI and satellites, Edmondson-Steiner (ES) grade, treatment modality, and Barcelona Clinic Liver Cancer (BCLC) stage. Statistical significance was set at p < 0.05. All statistical analyses were performed using R software, version 4.3.1.

## Results

### Study search and selection

**Figure 1:**
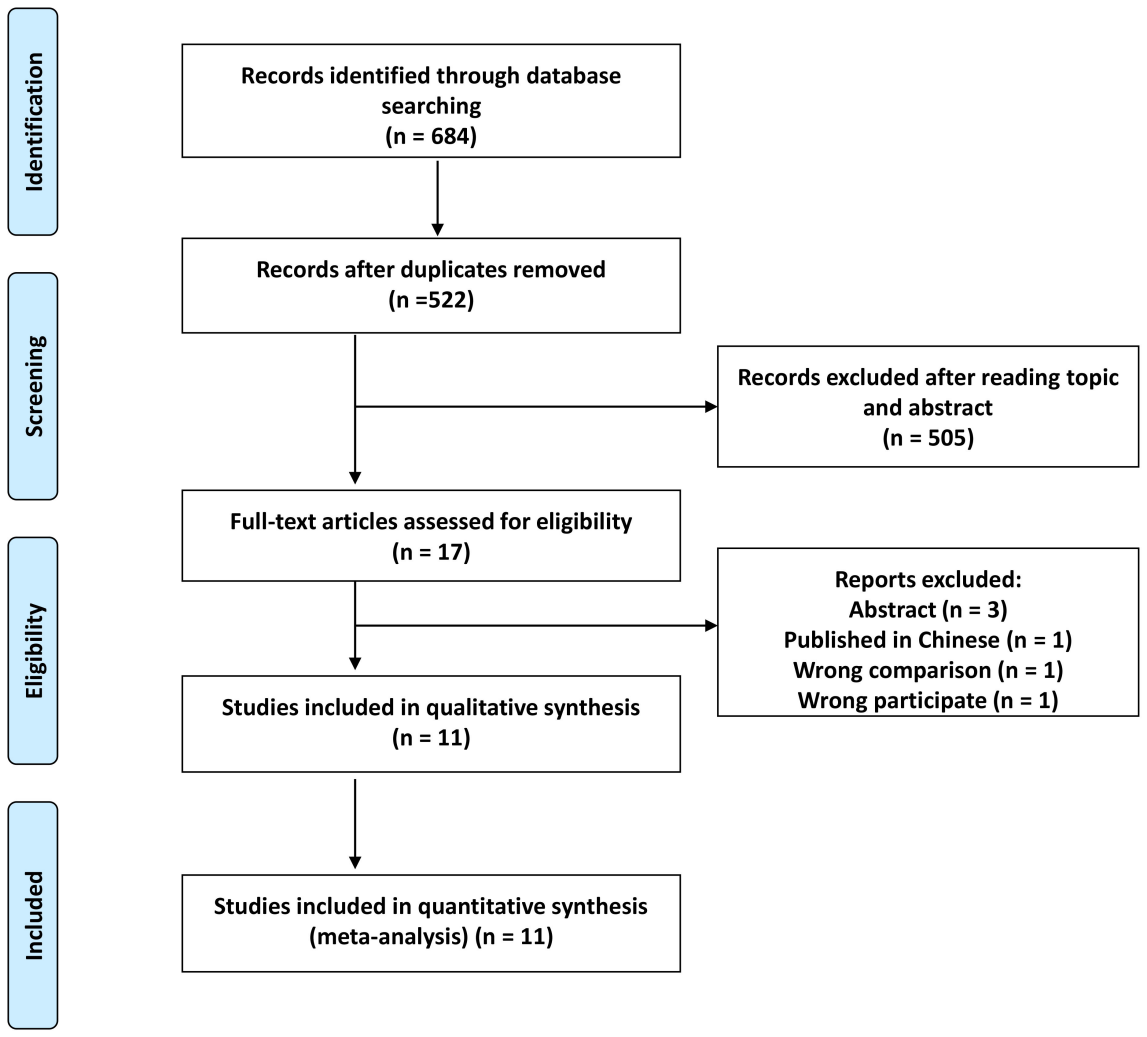
Flow chart of literature search.

A comprehensive search initially yielded 684 records, from which 162 duplicates were removed, resulting in 522 unique records. Subsequent screening of titles and abstracts led to the exclusion of 505 studies, leaving 17 articles for further scrutiny. After applying predetermined criteria, six articles were further excluded, resulting in 11 studies eligible for inclusion in our meta-analysis (**Figure 1**) (18, 19, 22–30).

### Study characteristics

Our analysis incorporated 11 studies, including two RCTs and nine retrospective studies, with a total of 3,219 patients. Among these, eight studies utilized propensity score matching (PSM) to derive outcomes (22–30). Five studies explicitly excluded patients who had received alternative adjuvant therapies, such as TACE or TKI monotherapy (23–25, 28, 29), whereas another five studies included varying proportions of patients who had undergone these additional treatments (18, 22, 26, 27, 30). Notably, one study investigated the efficacy of adjuvant ICIs in patients with HCC who had undergone either liver resection or radiofrequency ablation (18); however, our analysis exclusively focused on patients who underwent liver resection. The adjuvant therapies examined in these studies ranged from combinations such as TACE+ICIs and ICIs combined with lenvatinib or other TKIs to atezolizumab in conjunction with bevacizumab. **Table 1** and **Supplementary Material S2** provide detailed characteristics of the included studies, including those employing PSM. Quality assessment utilizing the NOS rated five studies at 7 points, four studies at 8 points, and one study at 9 points (**Supplementary Material S3**). The RCT conducted by Wang et al. was appraised as high risk in two blinded domains and low risk in the remainder (**Supplementary Material S4**).

**Table 1:** Characteristics of included studies.

Study	Group	Adjuvant therapy	Sample size	Age (year)	Gender M/F	HBV Y/N	Cirrhosis Y/N	Child-Pugh class A/B	AFP (ng/ml)	BCLC stage A/B/C	Tumor size (cm)	Tumor number S/Multiple	MVI Y/N	PVTT Y/N	Tumor satellite Y/N	ES grade I-II/III-IV	Survival months
Xu	ICI	ICIs alone or with TACE (33.9%)	109	56.7 ± 12	93/16	82/27	65/44	100/9	40 (>400) 69 (≤400)	NR	66 (>5) 43 (≤5)	71/38	35/74	NR	33/76	NR	mOS: 35.1 mRFS: 29.6
2024	No ICI	TACE (43.4%) or Active surveillance	518	56.2 ± 11	439/79	427/91	483/35	448/70	198 (>400) 320 (≤400)	NR	295 (>5) 223 (≤5)	348/170	274/244	NR	91/427	NR	mOS: 37.1 mRFS: 19.4
Wang	ICI	Sintilimab	99	53.0 (48.0–61.0)^ζ^	85/14	70/29	44/55	99/0	40 (>400) 59 (≤400)	99/0/0	58 (>5) 41 (≤5)	87/12	99/0	0/99	NR	54/45	mOS: Not reached mRFS: 27.7
2024	No ICI	Active surveillance	99	54.0 (49.0–61.0)^ζ^	83/16	75/24	56/43	99/0	35 (>400) 64 (≤400)	99/0/0	51 (>5) 48 (≤5)	86/13	99/0	0/99	NR	58/41	mOS: Not reached mRFS: 15.5
Ouyang	ICI	Camrelizumab + apatinib	111	28 (>60) 83 (≤60)	95/16	100/11	46/65	106/5	40 (>400) 71 (≤400)	94/17/0	69(>5) 42 (≤5)	86/25	111/0	0/111	18/93	73/38	mOS: Not reached mRFS: Not reached
2024	No ICI	Active surveillance	276	119 (>60) 157 (≤60)	226/50	248/28	133/143	274/2	106 (>400) 170 (≤400)	240/36	157 (>5) 119(≤5)	233/43	276/0	0/276	38/238	175/101	mOS: Not reached mRFS: 11.7
Huang	ICI	TACE+ICIs	83	3 (>65) 80 (≤65)	76/7	77/6	NR	78/5	46 (>400) 37 (≤400)	49/13/21	12.0 (10.4–15)^#^	68/15	58/25	NR	34/49	19/64	mOS: Not reached mRFS: 11.7
2024	No ICI	TACE	211	24 (>65) 187 (≤65)	188/23	191/20	NR	197/14	132 (>400) 79 (≤400)	153/24/34	11.8 (10.2–14)^#^	182/29	98/113	NR	54/157	58/153	mOS: Not reached mRFS: 6.9
Yuan	ICI	TACE+ICIs	42	23 (>50) 19 (≤50)	37/5	36/6	23/19	38/4	23 (>400) 19 (≤400)	0/0/42	33 (>5) 9 (≤5)	15/27	37/5	42/0	NR	38/4	mOS: 24.5 mRFS: 12.76
2023	No ICI	TACE	48	21 (>50) 27 (≤50)	43/5	44/4	28/20	43/5	28 (>400) 20 (≤400)	0/0/48	38 (>5) 10 (≤5)	18/30	40/8	48/0	NR	39/9	mOS: 19.1 mRFS: 8.11
Yang	ICI	ICIs + Target therapies	38	50.5 (44.0–54.8)^ζ^	34/4	35/3	26/12	37/1	11.3 (4.1–169.7)^ζ^	4/6/25	3.6 (1.1–5.7)^ζ^	26 (<3) 12 (≥3)	16/22	NR	NR	30/8	mRFS: 22
2023	No ICI	Active surveillance	158	55.0 (47.0–63.0)^ζ^	138/20	148/10	82/76	146/12	39.6 (4.8–599.0)^ζ^	73/12/49	4.5 (2.6–6.6)^ζ^	133 (<3) 25 (≥3)	72/85	NR	NR	131/27	mRFS: 11
Ouyang	ICI	ICIs + Lenvatinib	52	13 (>60) 39 (≤60)	49/3	NR	21/31	49/3	19 (>400) 33 (≤400)	39/13/0	40 (>5) 12 (≤5)	36/16	32/20	NR	19/33	31/21	mOS: 26.4 mRFS: Not reached
2023	No ICI	Active surveillance	85	36 (>60) 49 (≤60)	66/19	NR	31/54	81/4	28 (>400) 20 (≤400)	69/16/0	72 (>5) 13 (≤5)	69/16	49/36	NR	29/56	64/21	mOS: 26.6 mRFS: 5.5
Qin	ICI	Atezolizumab + bevacizumab	293	NR	NR	NR	NR	NR	NR	NR	5.3 (3.3–8.0)^ζ^	266/27	178/115	22/271	NR	169/124	mRFS: Not reached
2023	No ICI	Active surveillance	292	NR	NR	NR	NR	NR	NR	NR	5.9 (3.5–9.0)^ζ^	260/32	176/116	17/275	NR	171/121	mRFS: Not reached
Li, L	ICI	ICIs with or without TKIs	85	50.0 ± 9.7	74/11	66/19	63/22	75/10	27 (≥ 400) 58 (<400)	44/18/23	6.9 ± 4.1	58/27	42/43	NR	20/65	NR	mOS: Not reached mRFS: 25.2
2023	No ICI	Active surveillance	432	53.3 ± 11.8	387/45	336/96	360/72	380/52	157 (≥ 400) 275 (<400)	266/94/72	6.8 ± 3.9	340/92	174/258	NR	78/354	NR	mOS: Not reached mRFS: 16.1
Li, J	ICI	ICIs with TKIs	47	12 (≥60) 35 (<60)	43/4	31/16	NR	NR	27 (≥ 400) 20 (<400)	NR	34 (>5) 13 (≤5)	35/12	NR	NR	24/23	14/33	NR
2023	No ICI	Active surveillance	47	12 (≥60) 35 (<60)	42/5	29/18	NR	NR	27 (≥ 400) 20 (<400)	NR	34 (>5) 13 (≤5)	34/13	NR	NR	24/23	13/34	NR
Wen	ICI	ICIs	47	49.26 ± 12.23	45/2	41/6	10/37	44/3	18 (>400) 29 (<400)	14/14/19	79.15 ± 32.21 mm	26/21	28/19	17/30	5/42	NR	mOS: Not reached mRFS: 17.67
2023	No ICI	Active surveillance	47	50.81 ± 13.02	41/6	40/7	18/29	44/3	19 (>400) 28 (<400)	15/11/21	76.54 ± 43.69 mm	23/24	31/16	18/29	10/37	NR	mOS: Not reached mRFS: 5.73

ICI, immune checkpoint inhibitor; TACE, transarterial chemoembolization; TKI, Tyrosine Kinase Inhibitor; NR, not reported; M, male; F, female; HBV, hepatitis virus B; Y, yes; N, no; AFP, alpha-fetoprotein; BCLC stage, Barcelona Clinic Liver Cancer; S, solitary; MVI, microvascular invasion; PVTT, portal vein tumor thrombus; ES, Edmondson-Steiner.

ζ: data are presented as median and inter-quartile range.

: data are presented as median and range.

### OS and RFS

**Figure 2:**
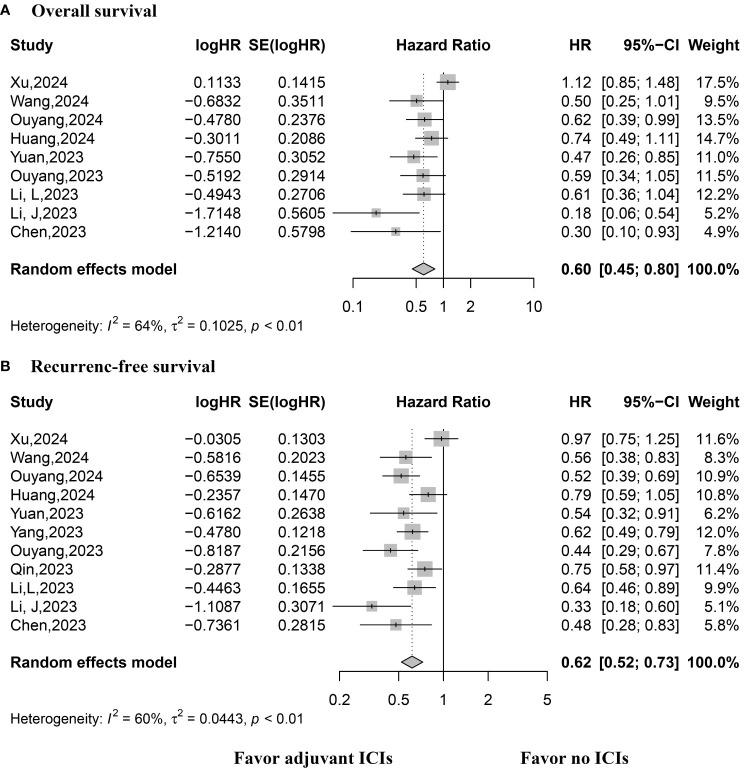
Forest plot of overall and recurrence-free survival rates. **(A)** Overall survival; **(B)** Recurrence-free survival.

Eight studies reported HRs for OS, necessitating the use of a random-effects model due to considerable variability. The pooled analysis demonstrated improved OS among patients who received adjuvant ICIs (HR, 0.60; 95% CI, 0.445–0.80; p < 0.0001; **Figure 2**). Similarly, all included studies reported HRs for RFS, with a random-effects model employed due to substantial heterogeneity. The synthesized results indicated improved RFS in patients receiving adjuvant ICIs (HR, 0.62; 95% CI, 0.52–0.73; p < 0.0001; **Figure 2**).

**Figure 3:**
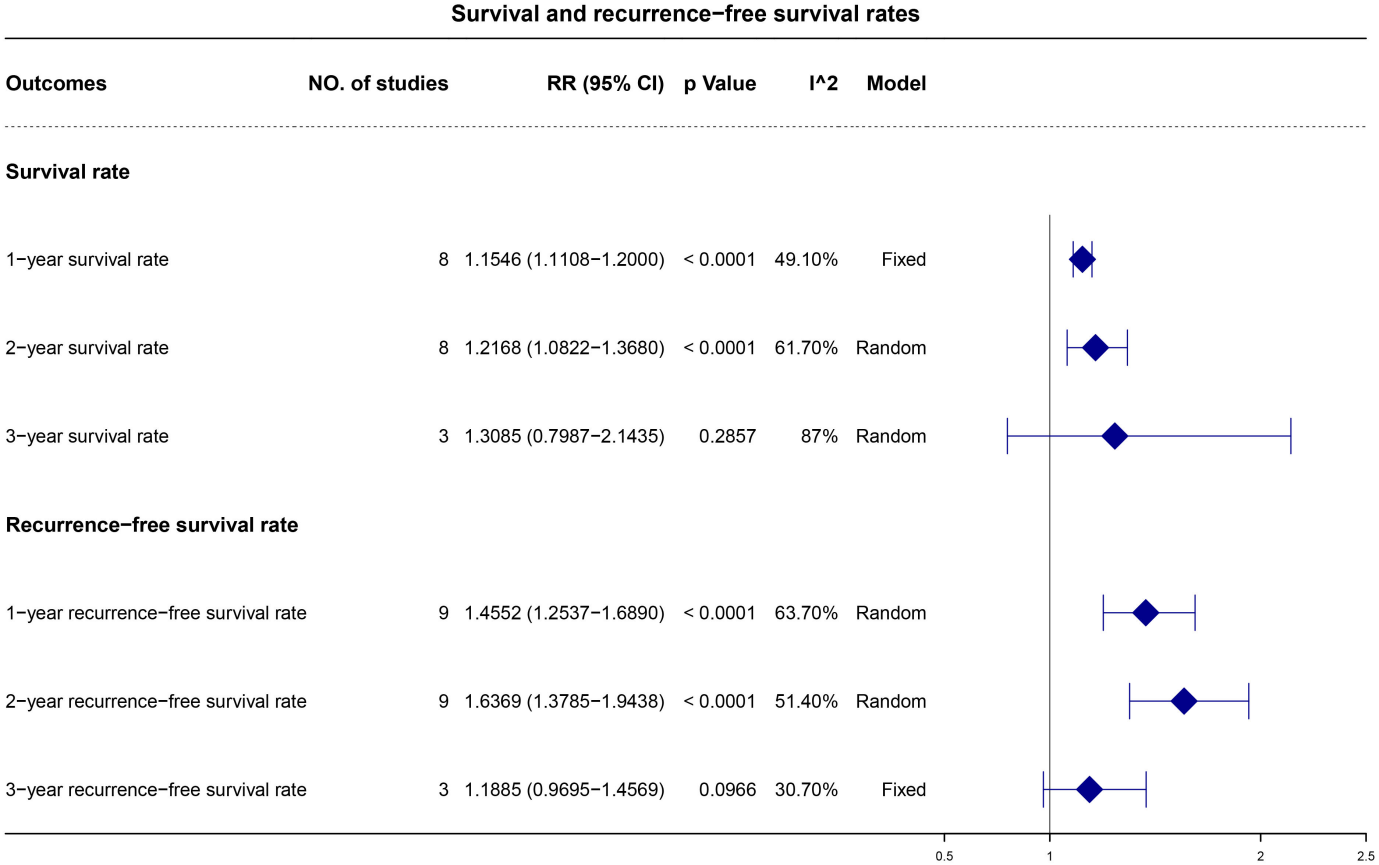
Forest plot for 1-, 2-, and 3-year overall and recurrence-free survival rates.

The 1-, 2-, and 3-year OS rates were reported in eight, eight, and three studies, respectively. A fixed-effects model was applied for the 1-year OS analysis, whereas a random-effects model was utilized for the 2- and 3-year OS analysis due to observed heterogeneity. The pooled results demonstrated higher 1- and 2-year OS rates with adjuvant ICI therapy (1-year RR, 1.15; 95% CI, 1.11–1.20; p < 0.0001 and 2-year RR, 1.22; 95% CI, 1.08–1.37; p < 0.0001), with similar 3-year OS rates (**Figure 3**).

The 1-, 2-, and 3-year RFS were reported in nine, nine, and three studies, respectively. A random-effects model was applied, except for the 3-year RFS analysis, which exhibited low heterogeneity. The synthesized data indicated higher 1- and 2-year RFS rates with adjuvant ICI therapy (1-year RR, 1.46; 95% CI, 1.25–1.69 and p < 0.0001; 2-year RR, 1.64; 95% CI, 1.38–1.94; p < 0.0001), with comparable 3-year RFS rates (**Figure 3**).

### AEs

**Figure 4:**
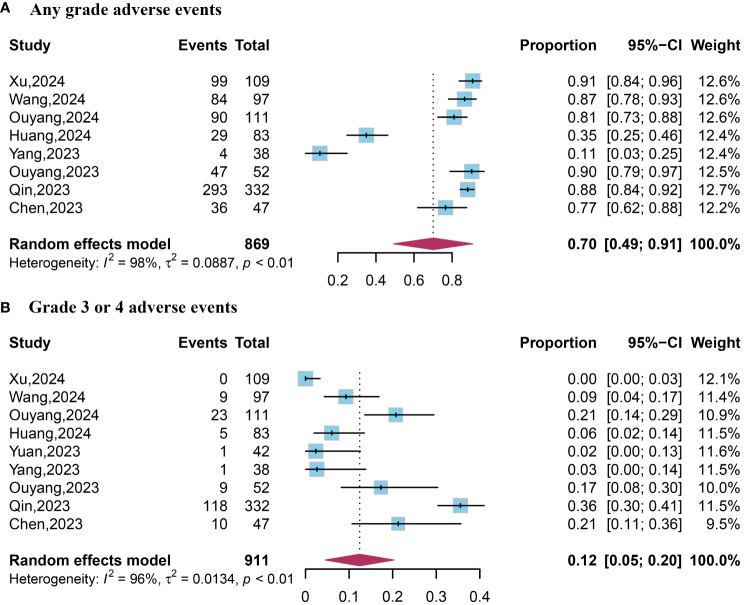
Forest plot of any grade and grade 3 or 4 adverse events. **(A)** Any grade adverse events; **(B)** Grade 3 or 4 adverse events.

Data on AEs of any grade were available from eight studies. The pooled analysis revealed an occurrence rate of 0.70 (95% CI, 0.49–0.91) for AEs of any grade (**Figure 3**). Data on grade 3 or 4 AEs were extracted from five studies. The pooled data reported an occurrence rate of 0.12 (95% CI, 0.05–0.20) for grade 3 or 4 AEs (**Figure 4**).

### Sensitivity analysis and publication bias

Sensitivity analysis findings are presented in **Supplementary Material S5**. Funnel plots for OS and RFS exhibited asymmetry, with Egger tests indicating significant publication bias (**Supplementary Material S6**). To address this bias, the trim and fill method was used to identify its potential influence on the results. We identified four studies for adjustment in both OS and RFS analysis (**Supplementary Material S7**). Subsequent forest plots based on the adjusted data revealed that the results for OS may have been impacted by publication bias, whereas those for RFS remained unaffected (**Supplementary Material S8**).

### Subgroup analysis

**Figure 5:**
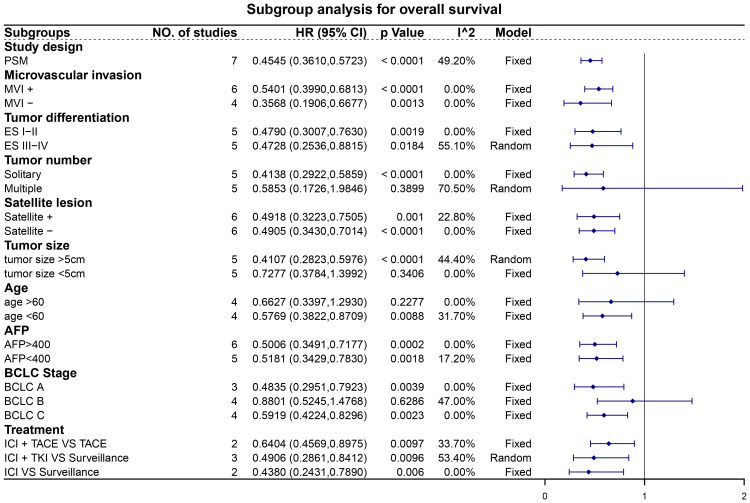
Subgroup analysis for overall survival. Abbreviations: PSM, propensity score matching; MVI, microvascular invasion; ES, Edmondson-Steiner; AFP, alpha-fetoprotein; BCLC, Barcelona Clinic Liver Cancer; ICI, immune checkpoint inhibitor; TACE, transarterial chemoembolization; TKI, tyrosine kinase inhibitor.

**Figure 6:**
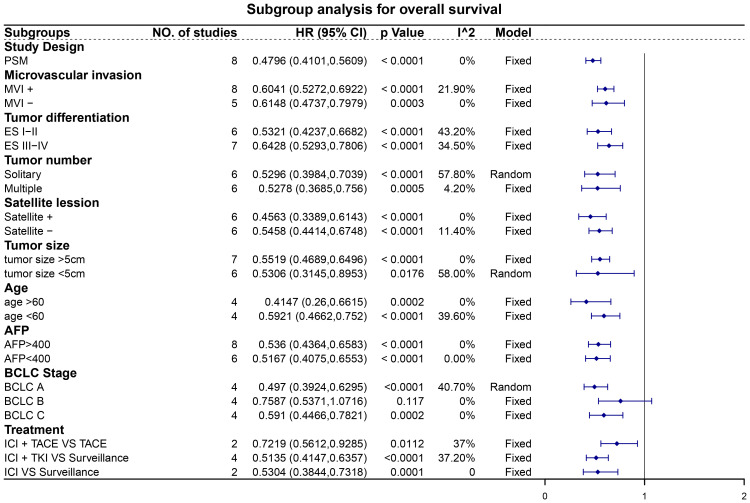
Subgroup analysis for recurrence-free survival. Abbreviations: PSM, propensity score matching; MVI, microvascular invasion; ES, Edmondson-Steiner; AFP, alpha-fetoprotein; BCLC, Barcelona Clinic Liver Cancer; ICI, immune checkpoint inhibitor; TACE, transarterial chemoembolization; TKI, tyrosine kinase inhibitor.

Subgroup analyses revealed noteworthy findings (**Figures 5**, **6**). Among the studies employing PSM, pooled data demonstrated that patients receiving adjuvant ICIs exhibited improved OS and RFS rates, with HRs of 0.45 (95% CI, 0.36–0.57; p < 0.0001) for OS and 0.48 (95% CI, 0.41–0.56; p < 0.0001) for RFS. Additionally, within the subgroups stratified according to high-risk recurrence factors, adjuvant ICIs notably improved OS among patients with MVI, ES grade III-IV, satellite lesions, tumor size > 5 cm, alpha-fetoprotein (AFP) levels > 400 ng/mL, and HCC categorized under BCLC stage C. Similarly, improvements in RFS rates were observed among patients with MVI, ES grade III-IV, multiple tumors, satellite lesions, tumor size > 5 cm, AFP levels > 400 ng/mL, and BCLC stage C HCC, regardless of whether adjuvant ICIs were combined with TACE and TKIs.

## Discussion

This meta-analysis highlights the potential of adjuvant ICI therapy following resection to improve both OS and RFS in patients with HCC exhibiting high-risk recurrence factors, with manageable AEs. Our study represents a pioneering endeavor to evaluate the efficacy and safety of adjuvant ICIs in patients with HCC exhibiting high-risk recurrence factors. The findings, bolstered by a statistically robust sensitivity analysis, provide credible evidence of the prognostic benefits associated with adjuvant ICI therapy in HCC cases. Subgroup analyses, particularly those utilizing PSM, revealed consistent findings, further reinforcing this conclusion. Moreover, these analyses, focusing on diverse tumor recurrence risk factors, underscore the potential of ICIs to ameliorate prognosis in patients with HCC exhibiting varied high-risk recurrence factors (10).

Early HCC recurrence often indicates tumors associated with heightened recurrence risks (31). Aggressive treatment of residual tumor cells could potentially enhance both RFS and OS, considering that occult micrometastases are present at initial HCC diagnosis. Several high-quality RCTs have investigated the efficacy and safety of ICIs, either alone or in combination with TKIs, for managing unresectable HCC (16, 17, 32–35). These trials have demonstrated that ICI therapy, alone or in combination with TKIs, yields comparable or superior prognoses compared with sorafenib treatments. ICIs function by reactivating effector CD4+ and CD8+ T cell functions via immune checkpoint inhibition, whereas TKIs optimize vascularization to enhance drug delivery and foster more robust tumor immune surveillance, potentially resulting in a synergistic effect in combination therapies. Consequently, ICIs, with or without TKIs, hold promise for targeting residual liver tumor cells (36). Furthermore, AEs appear less frequent with adjuvant therapies compared with first-line treatments for unresectable HCC, indicating a favorable safety profile for adjuvant ICIs (15–17, 32).

Despite variations in tumor characteristics and treatment modalities observed across the included studies, adjuvant ICI therapy consistently yielded improved prognoses. Nonetheless, the optimal adjuvant treatment strategy remains uncertain, emphasizing the need for tailored treatment selection based on individual patient characteristics, disease stage, and treatment response. Moreover, vigilant monitoring and adjustment as needed are crucial to optimize outcomes (37).

Our analysis is not without limitations. First, the number of included studies was modest, and most studies were retrospective. Second, although our subgroup analyses were extensive, their findings should be interpreted cautiously due to sample size limitations. Finally, publication bias was observed. These limitations warrant additional high-quality research to corroborate our findings.

## Conclusion

Adjuvant ICIs have demonstrated the potential to improve OS and RFS rates in patients with HCC exhibiting high-risk recurrence factors, with manageable AEs. However, additional high-quality research is needed to strengthen these findings.

## Data availability statement

The original contributions presented in the study are included in the article/**Supplementary Material**. Further inquiries can be directed to the corresponding authors.

## Author contributions

LH: Conceptualization, Data curation, Formal analysis, Investigation, Methodology, Software, Visualization, Writing – original draft, Writing – review & editing. YK: Conceptualization, Data curation, Formal analysis, Investigation, Methodology, Software, Visualization, Writing – original draft, Writing – review & editing. YQ: Funding acquisition, Validation, Writing – review & editing. AW: Funding acquisition, Validation, Writing – review & editing.
