# Supplementary material for: Can adjuvant immune checkpoint inhibitors improve the long-term outcomes of hepatocellular carcinoma with high-risk recurrent factors after liver resection? A meta-analysis and systematic review
Source: Front Oncol. 2024 May 24;14:1374262. doi: 10.3389/fonc.2024.1374262 (PMC11162111; doi:10.3389/fonc.2024.1374262)

Supplementary materials S1 Search strategy

| Databases | Search strategy | Number of records |
| --- | --- | --- |
| Pubmed | **#1 (((adjuvant) OR (postoperation)) OR (postoperative)) OR (****postresection)**  **#2 (((((immunotherapy) OR (immune checkpoint inhibitors)) OR (****immunological therapy)) OR (pd-1)) OR (pd-l1)) OR (****ctla-4)**  **#3 ((hepatocellular carcinoma[MeSH Terms]) OR (hepatocellular carcinoma)) OR (hcc)**  **#4 (((liver resection) OR (hepatic resection)) OR (hepatectomy)) OR (hepatectomy[MeSH Terms])**  #5 #1 AND #2 AND #3 AND #4 | 1,464,301  519,711  159,046  77,082  208 |
| Embase | #1 adjuvant:ti,ab,kw OR postoperation:ti,ab,kw OR postoperative:ti,ab,kw OR postresection:ti,ab,kw  #2 immunotherapy:ti,ab,kw OR 'immune checkpoint inhibitors':ti,ab,kw OR 'immunological therapy':ti,ab,kw OR 'pd 1':ti,ab,kw OR 'pd l1':ti,ab,kw OR 'ctla 4':ti,ab,kw  #3 'hepatocellular carcinoma':ti,ab,kw OR hcc:ti,ab,kw OR 'liver cancer'/exp  #4 'liver resection':ti,ab,kw OR 'hepatic resection':ti,ab,kw OR hepatectomy:ti,ab,kw OR 'hepatectomy'/exp  #5 #1 AND #2 AND #3 AND #4 | 1,187,920  286,802  367,875  82,234  193 |
| Web of Science | #1 TS=(adjuvant) OR TS=(postoperation) OR TS=(postoperative) OR TS=(postresection)  #2 TS=(immunotherapy) OR TS=(immune checkpoint inhibitors) OR TS=(immunological therapy) OR TS=(pd-1) OR TS=(pd-l1) OR TS=(ctla-4)  #3 TS=(hepatocellular carcinoma) OR TS=(hcc)  #4 TS=(liver resection) OR TS=(hepatic resection) OR TS=(hepatectomy)  #5 #1 AND #2 AND #3 AND #4 | 721,809  261,746  194,882  67,730  187 |
| Cochrane Library | #1 (adjuvant):ti,ab,kw OR (postoperation):ti,ab,kw OR (postoperative):ti,ab,kw OR (postresection):ti,ab,kw  #2 (immunotherapy):ti,ab,kw OR (immune checkpoint inhibitors):ti,ab,kw OR (immunological therapy):ti,ab,kw OR (PD-1):ti,ab,kw OR (PD-L1):ti,ab,kw OR (CTLA-4):ti,ab,kw  #3 MeSH descriptor:[Carcinoma, Hepatocellular] explode all trees  #4 (hepatocellular carcinoma):ti,ab,kw OR (hcc):ti,ab,kw  #5 #3 or #4  #6 (liver resection):ti,ab,kw OR (hepatic resection):ti,ab,kw OR (hepatectomy):ti,ab,kw  #7 MeSH descriptor:[Hepatectomy] explode all trees  #8 #6 or #7  #9 #1 AND #2 AND #5 AND #8 | 202,560  42,309  2,715  6,865  6,865  5,957  952  5,957  96(trials) |

Supplementary materials S2 Characteristics of PSM studies

| Study | Group | Adjuvant therapy | Sample size | Age (year) | Gender M/F | HBV Y/N | Cirrhosis Y/N | Child-Pugh class A/B | AFP (ng/ml) | BCLC stage A/B/C | Tumor size (cm) | Tumor number S/Multiple | MVI Y/N | PVTT Y/N | Tumor satellite Y/N | ES grade I-II/III-IV | Survival months |
| --- | --- | --- | --- | --- | --- | --- | --- | --- | --- | --- | --- | --- | --- | --- | --- | --- | --- |
| Xu | ICI | ICIs alone or with TACE (33.9%) | 99 | 56.5 ± 12 | 84/15 | 74/25 | 63/36 | 90/9 | 36 (>400) 63 (≤400) | 0/53/46 | 62 (>5) 37 (≤5) | 61/38 | 33/66 | NR | 27/72 | NR | mOS: 35.1 mRFS: 29.6 |
| 2024 | No ICI | TACE (43.4%) or Active surveillance | 99 | 56.7 ± 11 | 87/12 | 77/22 | 58/41 | 92/7 | 36 (>400) 63 (≤400) | 0/27/72 | 60 (>5) 39 (≤5) | 59/40 | 27/72 | NR | 32/67 | NR | mOS: 27.8mRFS: 19.3 |
| Wang | ICI | Sintilimab | 99 | 53.0 (48.0–61.0)^ζ^ | 85/14 | 70/29 | 44/55 | 99/0 | 40 (>400) 59 (≤400) | 99/0/0 | 58 (>5) 41 (≤5) | 87/12 | 99/0 | 0/99 | NR | 54/45 | mOS: Not reached mRFS: 27.7 |
| 2024 | No ICI | Active surveillance | 99 | 54.0 (49.0–61.0)^ζ^ | 83/16 | 75/24 | 56/43 | 99/0 | 35 (>400) 64 (≤400) | 99/0/0 | 51 (>5) 48 (≤5) | 86/13 | 99/0 | 0/99 | NR | 58/41 | mOS: Not reached mRFS: 15.5 |
| Ouyang | ICI | Camrelizumab + apatinib | 99 | 28 (>60) 71 (≤60) | 84/15 | 90/9 | 41/58 | 98/1 | 39 (>400) 60 (≤400) | 87/12 | 62 (>5) 37 (≤5) | 83/16 | 99/0 | 0/99 | 15/84 | 66/33 | mOS: Not reached mRFS: Not reached |
| 2024 | No ICI | Active surveillance | 172 | 56 (>60) 116 (≤60) | 146/26 | 154/18 | 73/99 | 171/1 | 61 (>400) 111 (≤400) | 151/21 | 104 (>5) 68 (≤5) | 144/28 | 172/0 | 0/172 | 22/150 | 113/59 | mOS: Not reached mRFS: 9.3 |
| Huang | ICI | TACE+ICIs | 77 | 3 (>65) 74 (≤65) | 70/9 | 72/5 | NR | 72/5 | 44 (>400) 33 (≤400) | 48/13/16 | 12.0 (10.4-15.0)^#^ | 64/13 | 52/25 | NR | 29/48 | 18/59 | mOS: Not reached mRFS: 11.7 |
| 2024 | No ICI | TACE | 77 | 4 (>65) 73 (≤65) | 70/9 | 72/5 | NR | 71/6 | 32 (>400) 45 (≤400) | 46/9/22 | 12.0 (10.8-14.0)^#^ | 63/14 | 52/25 | NR | 31/46 | 14/63 | mOS: 12.6mRFS: 3.9 |
| Yang | ICI | ICIs + Target therapies | 33 | 50.0 (44.0-55.0) ^ζ^ | 30/3 | 31/2 | 22/11 | 33/0 | 12.6 (4.1-293.1) ζ | 4/6/20 | 3.8 (1.7-5.7) ^ζ^ | 25 (<3) 18 (≥3) | 16/17 | NR | NR | 27/6 | mRFS: 22 |
| 2023 | No ICI | No adjuvant | 33 | 49.0 (44.0-56.0) ^ζ^ | 30/3 | 30/3 | 18/15 | 28/5 | 45.9 (6.4-487.0) ζ | 8/3/20 | 5.5 (2.7-8.1) ^ζ^ | 25 (<3) 18 (≥3) | 18/15 | NR | NR | 26/7 | mRFS: 6 |
| Li, L | ICI | ICIs with or without TKIs | 74 | 50.6 ± 9.6 | 66/8 | 57/17 | 57/17 | 67/7 | 23 (≥ 400) 51 (<400) | 43/15/16 | 6.7 ± 4.2 | 53/21 | 34/40 | NR | 16/58 | NR | mOS: Not reached mRFS: 30.0 |
| 2023 | No ICI | Active surveillance | 148 | 50.6 ± 11.3 | 131/17 | 113/35 | 113/35 | 128/20 | 47 (≥ 400) 101 (<400) | 78/40/30 | 6.8 ± 3.9 | 106/42 | 69/79 | NR | 27/121 | NR | mOS: Not reached mRFS: 15.5 |
| Li, J | ICI | ICIs with TKIs | 47 | 12 (≥60) 35 (<60) | 43/4 | 31/16 | NR | NR | 27 (≥ 400) 20 (<400) | NR | 34 (>5) 13 (≤5) | 35/12 | NR | NR | 24/23 | 14/33 | NR |
| 2023 | No ICI | Active surveillance | 47 | 12 (≥60) 35 (<60) | 42/5 | 29/18 | NR | NR | 27 (≥ 400) 20 (<400) | NR | 34 (>5) 13 (≤5) | 34/13 | NR | NR | 24/23 | 13/34 | NR |
| Chen | ICI | ICIs | 47 | 49.26±12.23 | 45/2 | 41/6 | 10/37 | 44/3 | 18 (>400) 29 (<400) | 14/14/19 | 79.15±32.21 mm | 26/21 | 28/19 | 17/30 | 5/42 | NR | mOS: Not reached mRFS: 17.67 |
| 2023 | No ICI | Active surveillance | 47 | 50.81±13.02 | 41/6 | 40/7 | 18/29 | 44/3 | 19 (>400) 28 (<400) | 15/11/21 | 76.54±43.69 mm | 23/24 | 31/16 | 18/29 | 10/37 | NR | mOS: Not reached mRFS: 5.73 |

Abbreviation: ICI, immune checkpoint inhibitor; TACE, transarterial chemoembolization; TKI, Tyrosine Kinase Inhibitor; NR, not reported; M, male; F, female; HBV, hepatitis virus B; Y, yes; N, no; AFP, alphafetoprotein; BCLC stage, Barcerona clinical liver cancer stage; S, solitary; MVI, microvascular invasion; PVTT, portal vein tumor thrombus; ES, Edmondson-Steiner.

ζ: data are presented as median and inter-quartile range.

#: data are presented as median and range.

Supplementary materials S3 Newcastle-Ottawa Scale scores of included studies

| Study | Selection | | | | Comparability | | Outcome | |  | Total score |
| --- | --- | --- | --- | --- | --- | --- | --- | --- | --- | --- |
|  | Representativeness of the exposed cohort | Selection of the non-exposed cohort | Ascertainment of exposure | Demonstration of outcome |  |  | Assessment of outcome | Follow-up was long enough | Adequacy of follow up |  |
| Xu 2024 | * | * | * | * |  |  | * | * | * | 7 |
| Ouyang 2024 | * | * | * | * |  |  | * | * | * | 7 |
| Huang 2024 | * | * | * | * |  |  | * | * | * | 7 |
| Yuan 2023 | * | * | * | * | * | * | * | * | * | 9 |
| Yang 2023 | * | * | * | * |  |  | * | * | * | 7 |
| Ouyang 2023 | * | * | * | * | * | * | * |  | * | 8 |
| Qin 2023 | * | * | * | * | * | * | * | * | * | 8 |
| Li, L 2023 | * | * | * | * |  |  | * | * | * | 7 |
| Li, J 2023 | * | * | * | * | * | * | * |  | * | 8 |
| Wen 2023 | * | * | * | * | * | * | * |  | * | 8 |

Supplementary materials S4 Risk assessment of RCTs

| Study | Random sequence generation | Allocation concealment | Blinding of participants  and personnel | Blinding of outcome assessment | Incomplete outcome data | Selective reporting | Other bias |
| --- | --- | --- | --- | --- | --- | --- | --- |
| Wang 2024 | Low Risk | Low Risk | High Risk | High Risk | Low Risk | Low Risk | Low Risk |

Supplementary materials S5 Sensitivity analyses for overall survival and recurrence-free survival. A, overall survival; B, recurrence-free survival.


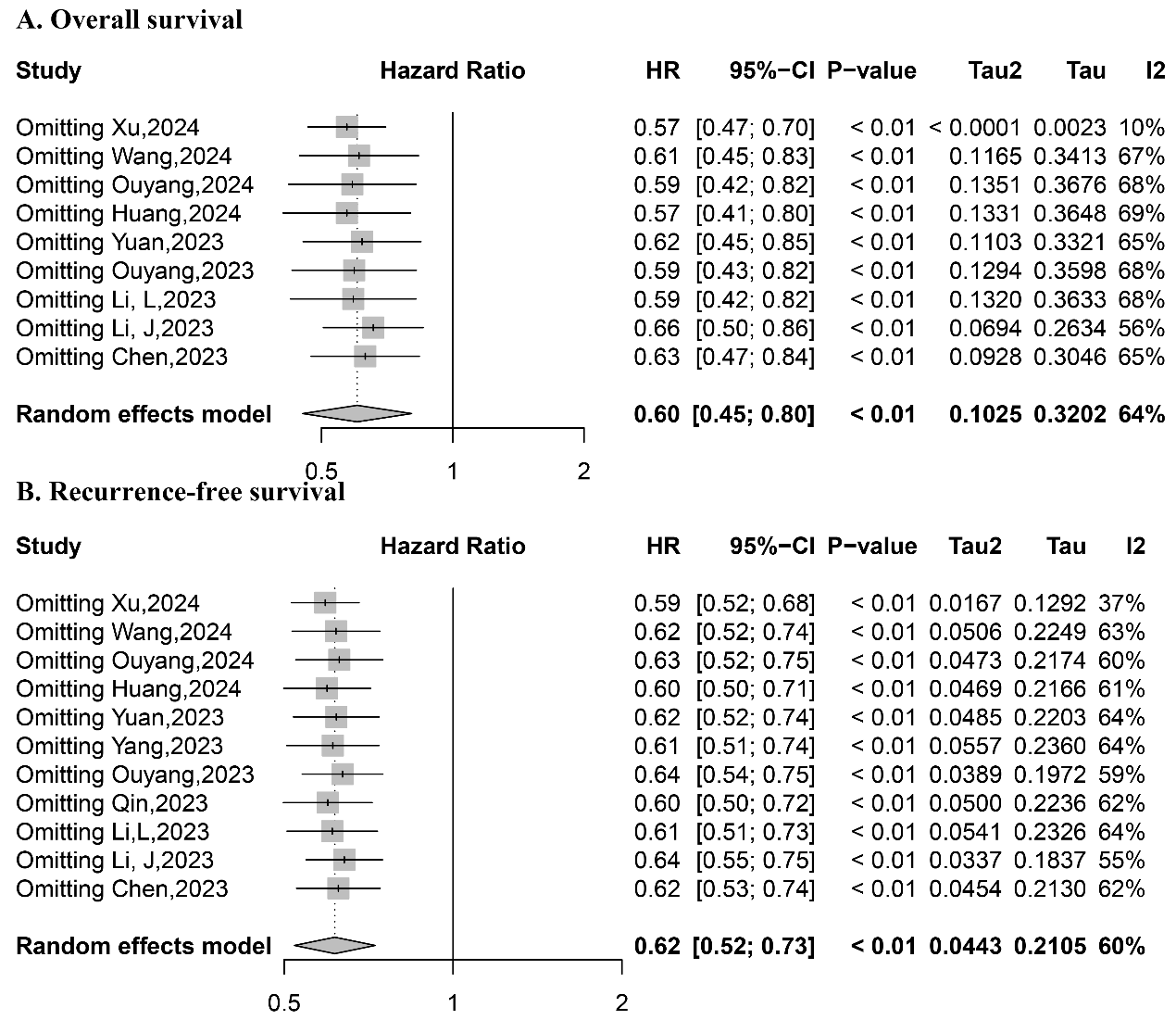


Supplementary materials S6 Funnel plot for overall survival and recurrence-free survival. A, overall survival; B, recurrence-free survival.


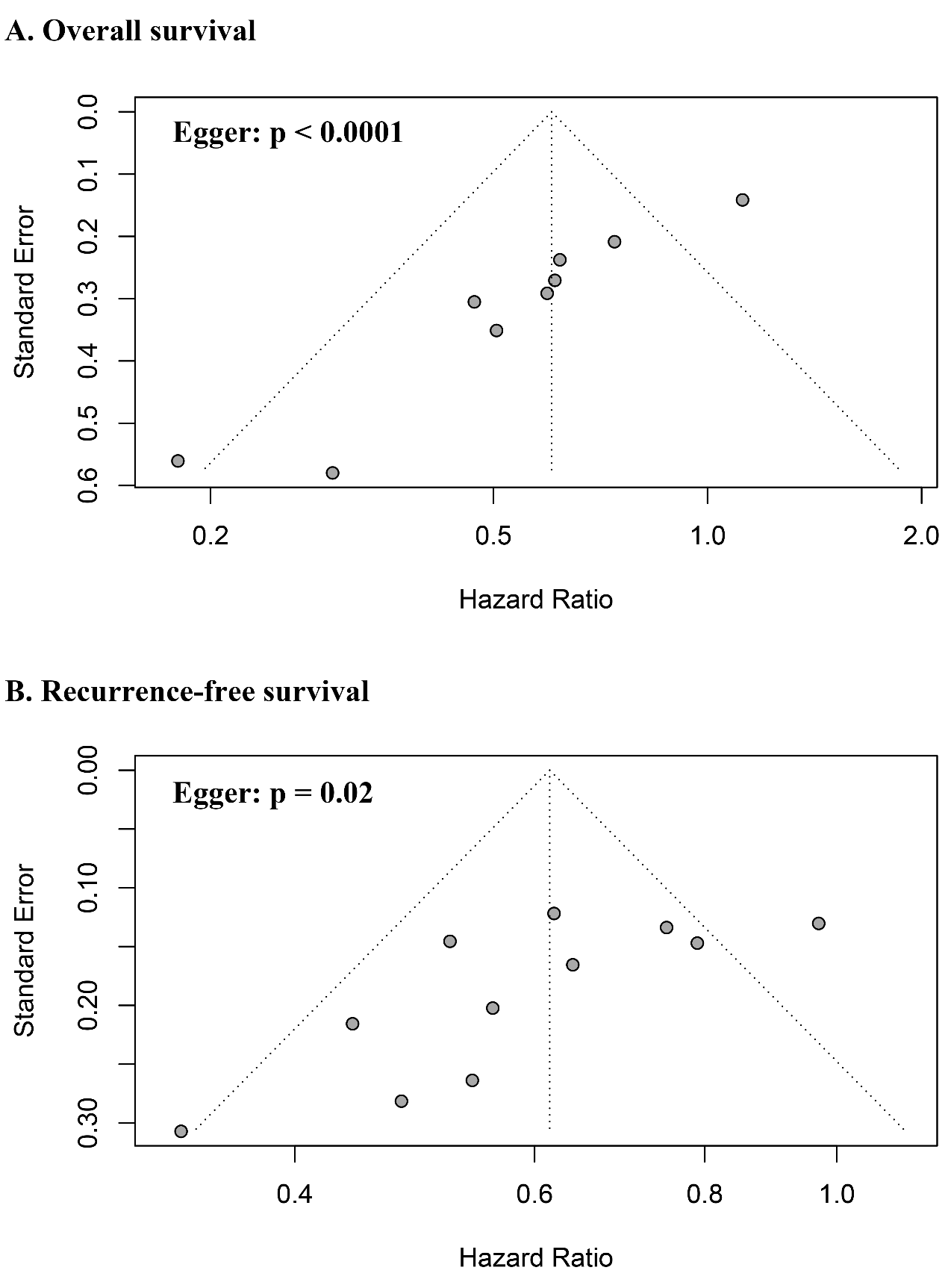


Supplementary materials S7 Funnel plot by after trimming and filling for overall survival and recurrence-free survival. A, overall survival; B, recurrence-free survival.


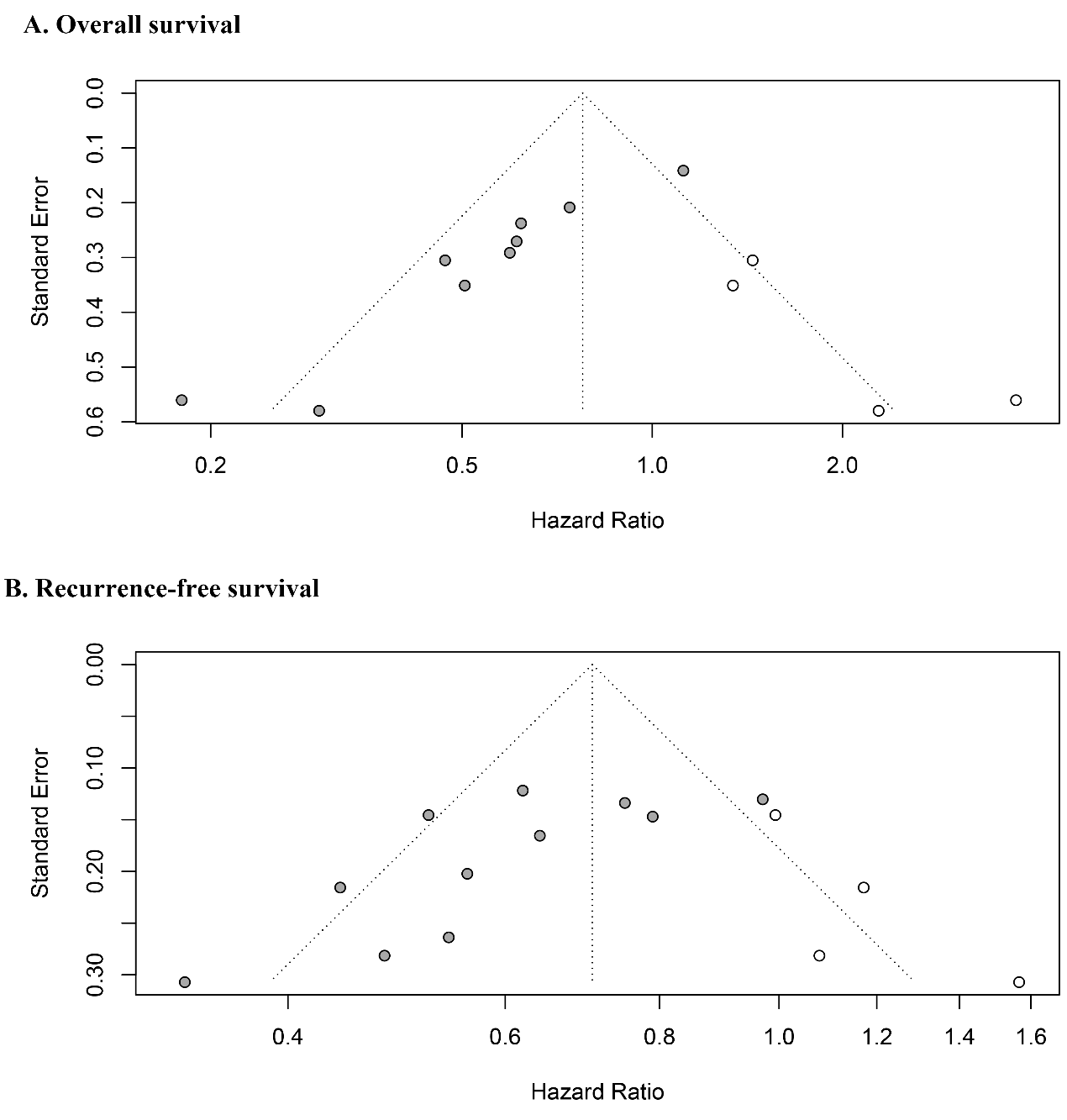


Supplementary materials S8 Forest plot by after trimming and filling for overall survival and recurrence-free survival. A, overall survival; B, recurrence-free survival.


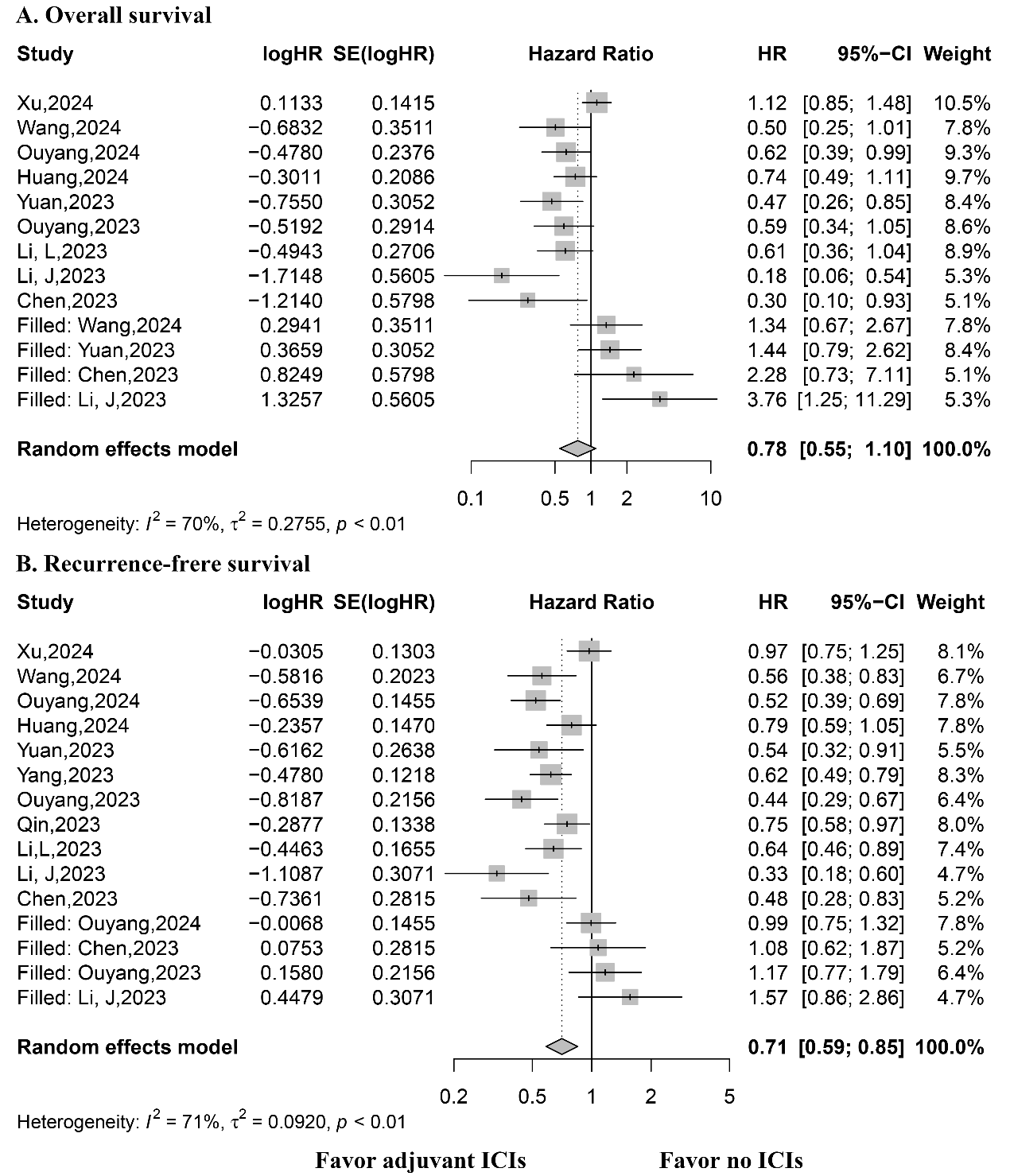

Supplement: Supplementary file 1 [file DataSheet_1.docx]
